# The TWIST1-centered competing endogenous RNA network promotes proliferation, invasion, and migration of lung adenocarcinoma

**DOI:** 10.1038/s41389-019-0167-6

**Published:** 2019-10-23

**Authors:** Wenjie Xia, Qixing Mao, Bing Chen, Lin Wang, Weidong Ma, Yingkuan Liang, Te Zhang, Gaochao Dong, Lin Xu, Feng Jiang

**Affiliations:** 1 Department of Thoracic Surgery, Nanjing Medical University Affiliated Cancer Hospital, Nanjing, China; 2 Jiangsu Key Laboratory of Molecular and Translational Cancer Research, Cancer Institute of Jiangsu Province, Baiziting 42, Nanjing, 210009 PR China; 3 The Fourth Clinical College of Nanjing Medical University, Nanjing, 210000 China; 4 Department of Hematology and Oncology, Department of Geriatric Lung Cancer Laboratory, The Affiliated Geriatric Hospital of Nanjing Medical University, Nanjing, Jiangsu China

**Keywords:** Non-small-cell lung cancer, Oncogenes

## Abstract

The proposed competing endogenous RNA (ceRNA) mechanism suggested that diverse RNA species, including protein-coding messenger RNAs and non-coding RNAs such as long non-coding RNAs, pseudogenes and circular RNAs could communicate with each other by competing for binding to shared microRNAs. The ceRNA network (ceRNET) is involved in tumor progression and has become a hot research topic in recent years. To date, more attention has been paid to the role of non-coding RNAs in ceRNA crosstalk. However, coding transcripts are more abundant and powerful than non-coding RNAs and make up the majority of miRNA targets. In this study, we constructed a mRNA-mRNA related ceRNET of lung adenocarcinoma (LUAD) and identified the highlighted TWIST1-centered ceRNET, which recruits SLC12A5 and ZFHX4 as its ceRNAs. We found that TWIST1/SLC12A5/ZFHX4 are all upregulated in LUAD and are associated with poorer prognosis. SLC12A5 and ZFHX4 facilitated proliferation, migration, and invasion in vivo and in vitro, and their effects were reversed by miR-194–3p and miR-514a-3p, respectively. We further verified that SLC12A5 and ZFHX4 affected the function of TWIST1 by acting as ceRNAs. In summary, we constructed a mRNA-mRNA related ceRNET for LUAD and highlighted the well-known oncogene TWIST1. Then we verified that SLC12A5 and ZFHX4 exert their oncogenic function by regulating TWIST1 expression through a ceRNA mechanism.

## Introduction

With approximately 2.1 million new cases diagnosed every year, lung cancer is the most common cause of cancer deaths worldwide and lung adenocarcinoma (LUAD) accounts for almost 40% of lung cancer deaths^1^. However, the pathogenesis of LUAD is still not very clear. Accordingly, investigating the mechanisms of tumorigenesis and progressive of LUAD could reveal crucial molecules which could serve as potential diagnostic biomarkers, or effective therapeutic targets.

A novel regulatory mechanism of RNA transcripts, known as competing endogenous RNA (ceRNA), was proposed by Pandolfi in 2011^2^. This proposed mechanism suggested that microRNAs (miRNAs), approximately 22 nucleotides in length, bind to miRNA response elements (MREs) in target mRNA molecules, resulting in decreased stability of the target mRNAs and repressed expression of the target RNA transcripts, such as mRNAs, long non-coding RNAs (lncRNAs), circular RNAs (circRNAs), pseudogenes like BRAF and HMGA1, which may modulate each other’s expression by competing for common miRNAs^3–8^. Complex crosstalk among ceRNAs has been found to occur in many different cancer types including LUAD^9–12^. In addition, since individual miRNAs are capable of targeting hundreds of genes, imbalance of gene expression could potentially propagate in the ceRNA networks (ceRNETs) through a cascade of co-regulated target RNAs and miRNAs that share targets, leading to mutual effects among distant components in the network. In the field of lung cancer, Sui et al. performed a computational analysis and identified a sponge interaction network among lncRNAs in human lung squamous cell carcinoma^13^. Until now, more attention has been paid to the role of non-coding RNAs in ceRNA crosstalk. However, coding transcripts are, in general, more abundant than lncRNAs, they show the highest conservation of miRNA binding sites, particularly in the 3′ UTR, and make up the majority of miRNA targets^14–17^. Thus, a reasonable inference to make from this is that mRNAs should exert the most influence in RNA competition.

In the present study, we constructed an mRNA-mRNA related ceRNA network containing 34,706 pairs of mRNA-mRNA communications based on target prediction algorithms provided by TargetScan, miRanda and Starbase websites and the co-expression relationships evaluated using expression data from The Cancer Genome Atlas (TCGA) (https://portal.gdc.cancer.gov/). Through this network, we identified the highlighted TWIST1-centered ceRNET, which recruits SLC12A5 and ZFHX4 as its ceRNAs.

TWIST1 is a well-known central regulator of epithelial-mesenchymal transition (EMT), which is widely expressed in human cancers^18–21^. High TWIST1 expression also relates to a high rate of metastasis, chemotherapeutic resistance and poor prognosis of patients with non-small cell lung cancer^22–25^. SLC12A5 is a potassium chloride cotransporter 2, which belongs to the solute carrier 12 (SLC12) family of potassium-chloride cotransporter proteins^26^. SLC12A5 was initially identified as an integral membrane KCl co-transporter that maintains chloride homeostasis in neurons^27,28^. Subsequent studies suggested that SLC12A5 is an oncogene which contributes to the progression and development of colorectal cancer and bladder urothelial carcinoma^29–32^. ZFHX4, a member of the zinc finger homeobox family of transcription factor proteins, was first identified in 1995^33^. ZFHX4 is a master regulator of CHD4 and SOX2 that regulates the glioblastoma tumor-initiating cell state, and its silencing results in decreased tumorigenesis and prolonged cancer-free survival of patients with glioblastoma^34^. However, the biological functions of SLC12A5 and ZFHX4 in LUAD are still unknown, also there are no studies on the ceRNA mechanism of these three genes,. Thus, in this study, we investigated whether SLC12A5 and ZFHX4 could exert their oncogenic function by regulating TWIST1 expression through a ceRNA mechanism in LUAD.

## Materials and methods

### Construction of ceRNA networks in LUAD

Based on the hypothesis of ceRNA theory, two principles were followed to screen the potential ceRNA pairs: sequence complementarity between miRNA and targeted mRNA (high miRNA regulatory similarity between mRNA pairs), and co-expression relationships between ceRNET components (positive co-expression relationships between mRNA-mRNA pairs and reverse co-expression relationships between mRNA and common miRNA).

For the principle of sequence complementarity, we predicted miRNA target sequences by three prediction programs, including TargetScan, miRanda and Starbase^35–37^. Intersectional miRNA and targeted mRNA pairs were selected. For the principle of co-expression relationships between ceRNA pairs (mRNA-mRNA) and their shared miRNAs, RNA sequencing data from individuals with LUAD (mRNA: 576 individuals; miRNA:495 individuals) were obtained from the TCGA database. Pearson correlation coefficient (*R*) of each candidate ceRNA pairs and its shared miRNAs were computed. All the candidate ceRNA pairs with *P*-adjusted < 0.05 (*R* < −0.09, *P*-adjusted < 0.05 for miRNA and targeted mRNA, *R* > 0.09, *P*-adjusted < 0.05 for mRNA and paired mRNA) were identified as ceRNA-ceRNA interactions. After assembling all identified ceRNA pairs, we generated the mRNA-mRNA related ceRNA network for LUAD. The intersection number of each mRNA in the network was recorded as node degree. The differentially expressed mRNAs were also screened out by the TCGA dataset. Expression differences were characterized by fold change and associated P-values. Fold change indicates the difference in expression of each RNA from LUAD to non-tumor tissues. Upregulated and down-regulated RNA were assigned fold changes >2 and <0.5, respectively. All P values were subject to false discovery rate (FDR) correction.

### Tissue samples and tissue microarrays

A total of 80 pairs of primary LUAD tissues and adjacent normal tissues (38 for real-time polymerase chain reaction (RT-PCR), 42 for immunohistochemical (IHC) staining) were collected from patients who had undergone surgery at the Department of Thoracic Surgery, Cancer Institute of Jiangsu Province, between 2016 and 2017 (Nanjing, China). All tumors and paired non-tumor tissues were confirmed by experienced pathologists. We obtained the written informed consent from all the patients. This study was approved by the Ethics Committee of the Nanjing Medical University Affiliated Cancer Hospital and was performed in accordance with the provisions of the Ethics Committee of Nanjing Medical University. Tissue microarray (TMA) was constructed as described previously^38^. Ninety-two pairs of LUAD tissues and adjacent nontumor tissues were used to construct the TMA.

### Cell culture, shRNA, 3′ UTR region, microRNA mimics, and inhibitor transfection

Human LUAD cell lines A549, H1299, PC9, H1975 and SPC-A-1 were purchased from Shanghai Institutes for Biological Science (Shanghai, China). A549, H1299, PC9 cells were maintained in RPMI 1640 (Hyclone, Logan, UT); H1975 and SPC-A-1 were maintained in DMEM medium (Hyclone, Logan, UT), supplemented with 10% fetal bovine serum (FBS, Invitrogen, USA) at 37 °C in the humidified atmosphere with 5% CO_2_.

LUAD cell lines at 50% confluency were transfected with 100 nM of either shRNAs (targeting TWIST1, SLC12A5, ZFHX4, GenePharma, Shanghai, China) or microRNA mimics/inhibitor (targeting miR-194-3p, miR-514a-3p, RiboBio, Guangzhou, China) using the Lipofectamine RNAimax reagent (Invitrogen, USA) according to the instructions provided by the manufacturer. Nonsense shRNA (sh-nc) and negative control mimic (miR-nc) were used as the respective controls. The shRNA sequences were listed in Supplementary Table 1. The 3′UTR regions (Sequence listed in Supplementary Table 2) of TWIST1, SLC12A5, ZFHX4 were synthesized and cloned into the expression vector pcDNA3.1 (Biogot technology, Nanjing, China). The final construct was verified by sequencing. Transfection of 3′-UTR regions was performed according to the Lipofectamine 3000 Reagent (Invitrogen, USA) protocol.

### RNA extraction and RT-PCR analyses

Total RNA was extracted from cell lines and tissues with Trizol reagent (Life Technologies, Scotland, UK) according to the manufacturer’s protocol. A 1.5-μg total RNA was reverse transcribed in a final volume of 20 μl using random primers under standard conditions using the PrimeScript RT Master Mix (Takara, Cat. #RR036A). After the RT reaction, the RT-PCR was performed using the SYBR Select Master Mix (Applied Biosystems, cat: 4472908) with 0.5 μl complementary DNA (cDNA) on ABI 7300 system (Applied Biosystems, Foster City, CA, USA). Mature microRNA levels were quantified using TaqMan microRNA Assay (Applied Biosystems). GAPDH, snRNA U6 were used as internal controls. Primers for microRNAs were acquired from RiboBio Co. Other primer sequences are listed in Supplementary Table 3.

### Protein extracts and western blot analysis

Cells were harvested and treated with lysis buffer (RIPA, KeyGEN) on ice, and protein concentration was determined using a BCA Kit (KeyGEN). Comparable amounts of extracts were loaded on SDS–PAGE gels and subjected to electrophoresis. After separation on the gel, proteins were transferred to a PVDF membrane. Membranes were blocked in 2% BSA in TBS-T for 1 h, and subsequently incubated overnight, at 4 °C, with antibodies against Anti-TWIST1(Abcam, Cambridge, UK; ab175430) and anti-ZFHX4 (Abcam, Cambridge, UK; ab57782) mouse monoclonal antibodies, anti-SLC12A5 rabbit polyclonal antibody (Abcam, Cambridge, UK; ab97502) or b-actin (Cell Signaling Technology, Danvers, MA, USA; 8H10D10). After washing in TBS-T, membranes were incubated with goat anti-rabbit or anti-mouse secondary antibodies (both from Abcam; 1:10,000), for 2 h at room temperature. Blots were visualized using ECL detection (Thermo Fisher Scientific, Waltham, MA, USA). All experiments were repeated at least three times, independently.

### IHC staining

Tissue sections were deparaffinized and rehydrated through graded alcohol. Endogenous peroxidase activity was blocked by incubation in 3% H_2_O_2_. Antigen retrieval was carried out with 0.01 M citrate buffer (pH 6.0) and microwave heat induction. Immunohistochemistry was performed on serial 2.5 μm thick tissue sections from the TMAs or the original blocks. Anti-TWIST1 and Anti-ZFHX4 mouse monoclonal antibodies and anti-SLC12A5 rabbit polyclonal antibody was used. Individual specimens were evaluated by two pathologists in a blind method, and those with scores of greater than 0.5 were defined as positive expression, less than or equal to 0.5 were negative expressions.

### Immunofluorescence double staining

Cells grown on coverslips were fixed with 4% paraformaldehyde, permeated with 0.3% Triton X-100 and blocked with 1% BSA. Then cells were in turn incubated with a mixture of two primary antibodies (rabbit anti-TWIST1, and mouse anti-SLC12A5/anti-ZFHX4) at 4 °C overnight, as well as a mixture of secondary antibodies (Alexa Fluor 488 and 594, 1:2000, Thermo Fisher Scientific) in the dark for 1 h. Nuclei were counterstained by 4′,6-diamidino-2-phenylindole (DAPI, Thermo Fisher Scientific).

### In vitro cell proliferation, migration, invasion assays, and xCELLigence system assays

Cell proliferation was examined using 5-ethynyl20deoxyuridine (EdU) assay kits (RiboBio) and Cell Counting Kit-8 (CCK-8, Roche Applied Science), following the research protocol.

For migration assays, A549 or H1299 treated cells (2.5 × 10^5^) were plated in the upper chamber of transwell assay inserts (Millipore, Billerica, MA, USA) containing 200 μl of serum-free RPMI 1640 with a membrane (8-mm pores). Then inserts were placed into the bottom chamber wells of a 24-well plate filled with conditioned medium. After 24 h of incubation, the cells on the filter surface were fixed with methanol, stained with crystal violet, and photographed with a digital microscopy. Cell numbers were calculated in five random fields for each chamber. For invasion assays, transfected cells (4 × 10^5^) were plated in the top chamber with a Matrigel-coated membrane (BD Biosciences) in 500-μl serum-free RPMI 1640 accompanied with 750 μl 10% FBS-1640 in the bottom chambers. After a 48-h incubation period, we determined the invasion function as mentioned previously in migration.

The CIM-plate16 contains 16 wells, each a modified Boyden chamber, which can be used independently but simultaneously to measure cell migration in real‐time through 8 μm pores of a polyethylene terephthalate membrane on to gold electrodes on the underside of the membrane using the xCELLigence system (ACEA Biosciences, San Diego, CA, USA). Experiments were set up according to the manufacturer’s instructions with the membrane uncoated (migration) or coated with growth-factor-reduced-matrigel (invasion) (BD BioSciences, Oxford, UK) (20 μl 1:40 diluted matrigel per well on the upper surface). Cell index (electrical impedance) was monitored every 15 min for the duration of the experiment. Traces show the average of quadruplicate wells.

### RNA immunoprecipitation (RIP)

The EZMagna RIP Kit (MilliporeSigma, USA) was used following the manufacturer’s protocol. A549 or H1299 cells were lysed in complete RIP lysis buffer, and the cell extract was incubated with magnetic beads conjugated with anti-Argonaute 2 (AGO2) or control anti-IgG antibody (MilliporeSigma, USA) for 6 h at 4 C. The beads were washed and incubated with Proteinase K to remove proteins. Finally, purified RNA was subjected to RT-PCR analysis.

### Luciferase reporter assay

The binding sites of 3′UTR regions and miRNAs were predicted by TargetScan. The different fragment sequences (listed in Table S3) were synthesized and then inserted into the pcDNA3.1 (+) and psiCHECK-2 vector (Promega). All vectors were verified by sequencing, and luciferase activity was assessed using the Dual Luciferase Assay Kit (Promega) according to the manufacturer’s instructions.

### Biotin-coupled miRNA capture

The biotin-coupled miRNA pull-down assay was performed as described previously by Zheng and colleagues^39^. Briefly, the 3′ end biotinylated miR-RNA mimic or control biotin-RNA (RiboBio, Guangzhou, China) was transfected into H1299 or A549 cells at a final concentration of 20 nmol/L for 1 day. The biotin-coupled RNA complex was pulled down by incubating the cell lysate with streptavidin-coated magnetic beads (Ambion, Life Technologies). The abundance of TWIST1, SLC12A5, and ZFHX4 in bound fractions was evaluated by RT-PCR analysis.

### In vivo animal model and lung metastasis

Thirty female BALB/c nude mice weighing 18–22 g were randomly assigned to six groups. H1299 cells were prepared as a suspension of 10^6^ cells in 200 μl saline and injected subcutaneously after transfected with sh-nc or shRNA. The tumor size was measured every 2 days with calipers. Six weeks after injection, the mice were sacrificed and the subcutaneous tumors were isolated and measured. The animal study was carried out according to the State Food and Drug Administration of China regulations on animal care. Animals were sorted only by treatment, there was no exclusion or inclusion of an animal was predetermined.

### Statistical analysis and software used

All statistical analyses were performed using SPSS version 19.0 software (SPSS Inc., Chicago, USA). Specific blinding or randomization method was not applied. The size of each experimental group was limited according with reproducibility and extent of difference; generally, small groups (3–4 independent individuals) were only considered for determination with an evident qualitative difference between groups. Student’s t-test and one-way ANOVA analysis were applied to evaluate differences between two or more groups. Chi-square analysis was used evaluate the relationship of TWIST1/ZFHX4/SLC12A5 in 42 IHC staining cases of LUAD. Survival curves were plotted using the Kaplan–Meier method, and differences between survival curves were tested using the log-rank test. *R* values and *P* values were generated by Pearson linear analysis to assess the co-expression of mRNAs and miRNAs. Perl program was used to generate miRNA-mRNA pairs from TargetScan, miRanda and starbase. Pearson correlation *R* value of each candidate ceRNA pair and the differentially expressed mRNAs were computed by R project. The ceRNA networks were visualized by Cytoscape 3.0.2^40^.

## Results

### Construction of ceRNA networks in LUAD

**Fig. 1:**
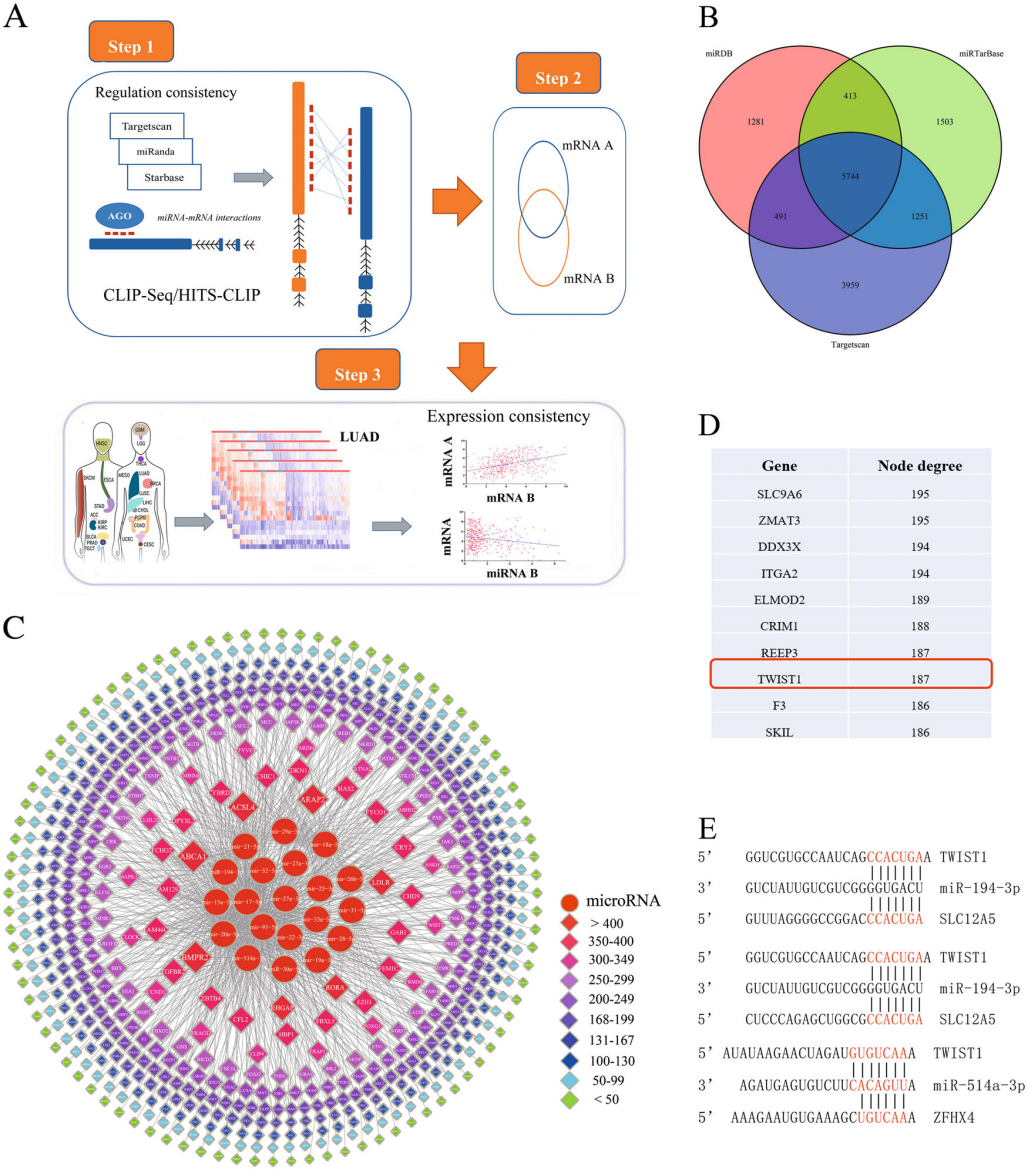
Construction of ceRNA networks in lung adenocarcinoma (LUAD). **a** The workflow for constructing and characterizing the miRNA-mediated mRNA related ceRNA network. Step1: The miRNA-target regulations were identified by integration of the CLIP-seq dataset with three prediction algorithms. Step2: mRNA-mRNA interactomes were enumerated based on the 5744 pairs of miRNA-mRNA. Step3: The gene expression profiles were collected from the TCGA database and the mRNA interactions were identified by considering the expression consistency. **b** 5744 pairs of miRNA-mRNA form the intersection predicted by TargetScan, miRanda and Starbase. **c** The landscape of mRNA-mRNA interaction networks. Its graphic visualization uses nodes to represent individual ceRNAs and edges to represent miRNA-mediated RNA-RNA interactions. The color bands which include nodes with similar node degree (number of interactions), have a size increases with the distance from the center. **d** The highlighted oncogene TWIST1 with a node degree of 187. **e** Predicted binding sites for miR-194-3p with TWIST1 (Position 378–384 of TWIST1 3′ UTR)/SLC12A5 (Position 1169–1176, 1636–1642 of SLC12A5 3′ UTR), and miR-514a-3p with TWIST1 (Position 2335–2342 of TWIST1 3′ UTR)/ZFHX4 (Position 1491–1497 of ZFHX4 3′ UTR)

A central tenet of our hypothesis is that trans-regulatory ceRNA crosstalk increased with the high miRNA regulatory similarity between mRNAs and their strong co-expression relationships. The construction of the mRNA-miRNA-mRNA regulatory landscape in LUAD was conducted in three steps. Figure 1a is the workflow for constructing and characterizing the miRNA-mediated mRNA related ceRNA network. In the first step, 5744 pairs of miRNA-mRNA were predicted by TargetScan, miRanda and Starbase based on sequence complementarity principle and formed the intersection (Fig. 1b, Supplementary data). In the second step, mRNA-mRNA interactomes were enumerated based on the 5,744 pairs of miRNA-mRNA. In the third and final step, the Pearson correlation coefficient of each candidate mRNA-mRNA ceRNA pairs and its shared miRNAs were computed. All the candidate ceRNA pairs with *P*-adjusted < 0.05 (*R* < −0.09, *P*-adjusted < 0.05 for miRNA and targeted mRNA, *R* > 0.09, *P*-adjusted < 0.05 for mRNA and paired mRNA) were identified as potential ceRNA-ceRNA interactions. As a result, 34,706 pairs of mRNAs were screened out as ceRNA landscape in LUAD (Fig. 1c, Supplementary data). The number of intersections of each mRNA in the network was defined as the node degree (Supplementary data).

We also applied the RNA sequencing data obtained from TCGA database to filter all the high-expressed genes in tumor tissues (*P*-adjusted < 0.05, Supplementary data). We contrasted the ceRNAs in the network with the highly expressed genes in tumor tissues and highlighted the TWIST1 with a node degree of 187 (Fig. 1d). Searching through the LUAD ceRNA network, we found the TWIST1-centered ceRNET, which recruits SLC12A5 and ZFHX4 as its ceRNAs (Fig. 1e).

### Validation of co-expression relationships

**Fig. 2:**
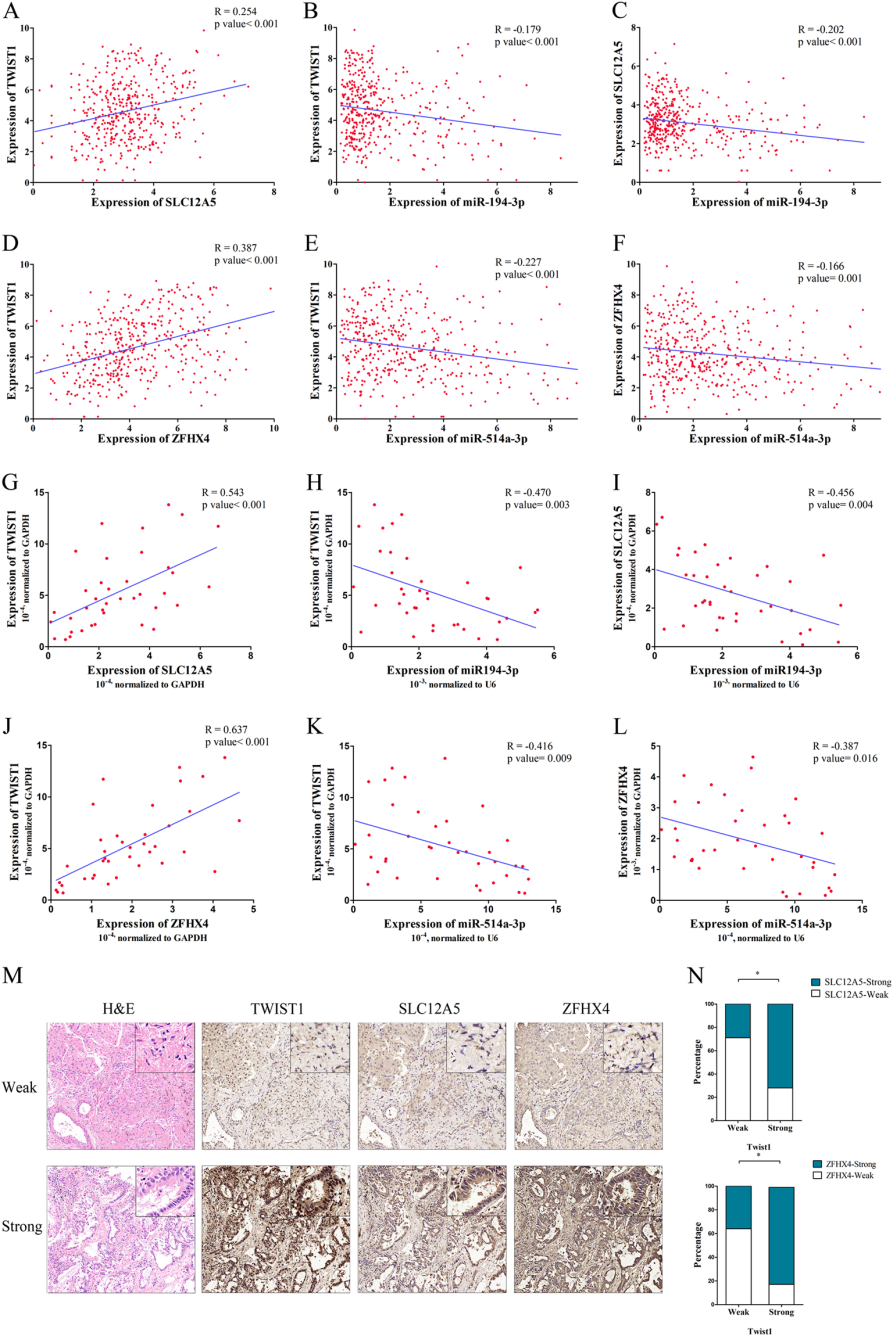
Validation of co-expression relationships in TCGA dabaset and LUAD tissues. **a**–**c** TWIST1 exhibited a positive correlation with that of SLC12A5 (**a**). Both TWIST1 (**b**) and SLC12A5 (**c**) exhibited a reverse correlation with that of miR-194-3p in TCGA dataset. **d**–**f** TWIST1 exhibited a positive correlation with that of ZFHX4 (**d**). Both TWIST1 (**e**) and ZFHX4 (**f**) exhibited a reverse correlation with that of miR-514a-3p in TCGA dataset. **g**–**l** RT-PCR analysis of 38 paired fresh LUAD patients’ tissue samples reached consistent results with those of TCGA database. **m** Immunohistochemical staining of TWIST1/SLC12A5/ZFHX4 in relatively the same range. **n** High expression of TWIST1 was correlated with high expression of SLC12A5 and ZFHX4 evaluated by immunohistochemical staining in 42 cases of LUAD. The data statistical significance is assessed by Pearson correlation (**a**–**l**) and Chi-square analysis (**n**). *P < 0.05, **P < 0.001

We further demonstrate the co-expression of these genes by extracting the expressions of the TWIST1-centered ceRNET from the TCGA dataset mentioned above. As shown in Fig. 2a-c, the expression of TWIST1 exhibited a positive correlation with that of SLC12A5 (Pearson’s *R* = 0.254, *P* < 0.001). Additionally, both TWIST1 and SLC12A5 exhibited a reverse correlation with that of miR-194-3p (TWIST1: *R* = −0.179, *P* < 0.001; SLC12A5: *R* = −0.202, *P* < 0.001). In addition, as shown in Fig. 2d–f, expression of TWIST1 showed a positive correlation with that of ZFHX4 (*R* = 0.387, *P* < 0.001). Also, both TWIST1 and ZFHX4 were reversely correlated with miR-514a-3p (TWIST1: *R* = −0.227, *P* < 0.001; ZFHX4: *R* = −0.166, *P* < 0.001).

The expression of the TWIST1-ceRNET components was also detected by RT-PCR analysis of 38 paired fresh LUAD patients’ tissue samples (tumor and adjacent normal lung tissue samples). We selected the tumor tissue samples to validate the expression relationship among the TWIST1-ceRNET components and obtained consistent results (Fig. 2g–l). IHC staining of TWIST1/SLC12A5/ZFHX4 was also performed in LUAD tissue samples from 42 cases of LUAD using serial sections to evaluate their co-expression in relatively the same range (Fig. 2m). Accordingly, SLC12A5 was highly expressed in 17 out of 24 TWIST1 strong tumor tissue samples, and in 5 out of 18 tumor tissue samples with weak TWIST1 expression; ZFHX4 was highly expressed in 19 out of 24 TWIST1 strong tumor tissues, and in 6 out of 18 tumor tissue samples with weak TWIST1 (Fig. 2n). The results above demonstrated the co-expression relationships between TWIST1-ceRNET in LUAD tissues.

### Overexpression of TWIST1, SLC12A5, and ZFHX4 correlate with more aggressive clinical characteristics and poor prognosis in patients with LUAD

**Fig. 3:**
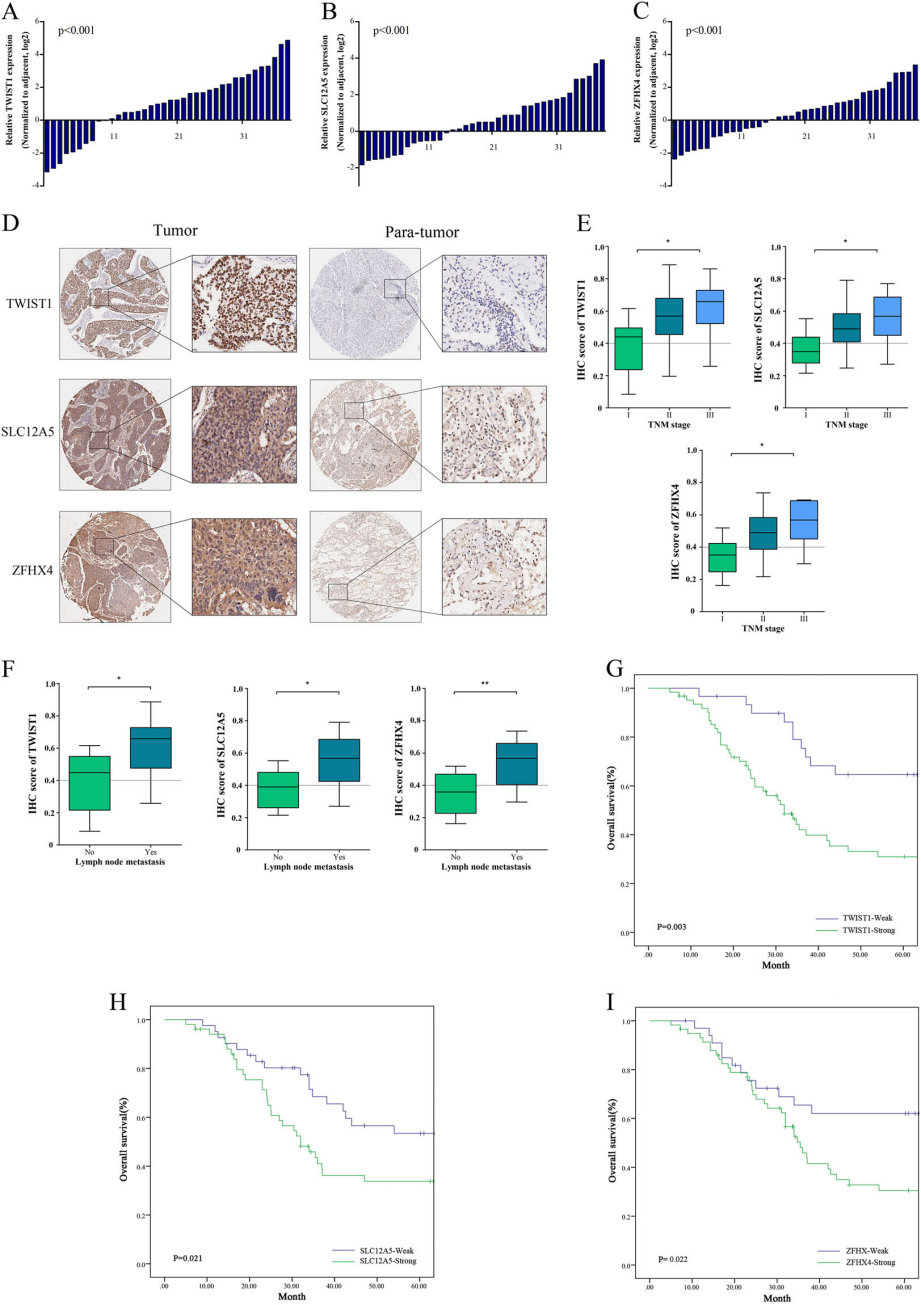
Overexpression of TWIST1, SLC12A5, and ZFHX4 correlate with more aggressive clinical characteristics and poor prognosis in patients with LUAD. **a**–**c** TWIST1 (**a**), SLC12A5 (**b**) and ZFHX4 (**c**) were significantly overexpressed in the 38 LUAD tissue samples evaluated by RT-PCR. **d** Immunohistochemical staining analysis of TWIST1/SLC12A5/ZFHX4 in LUAD tissue microarray containing 92 pairs of LUAD and matched non-tumor tissue samples. **e**, **f** High expression of the TWIST1-ceRNET components was significantly correlated with advanced TNM staging (**e**) and lymph node metastasis (**f**). **g**–**i** Kaplan–Meier Plotter indicated that patients with higher TWIST1 (**g**)/SLC12A5 (**h**)/ZFHX4 (**i**) expression had poor OS in 92 LUAD patients after surgery. Empty vector (EV), RNA control (miR-C) and sh-control (sh-C) were used as controls as the circumstances may require. The data statistical significances were assessed by Student’s *t*-test (**a**–**c**, **f**), one-way ANOVA analysis (**e**) and log-rank test (**g**–**l**). *P < 0.05, **P < 0.001

The results obtained by RT-PCR analysis of 38 pairs of LUAD tissue samples, shown in Fig. 3g–l, revealed that TWIST1, SLC12A5 and ZFHX4 were all significantly overexpressed in the LUAD tissue samples, with average up-regulation folds of 4.14 (*p* < 0.001), 3.71 (*p* < 0.001) and 3.68 (*p* < 0.001), respectively (Fig. 3a–c). We used our own LUAD tissue microarray containing 92 pairs of LUAD and matched non-tumor tissue samples with clinicopathologic information and long-time follow-up records to evaluate the clinical utility of TWIST1, SLC12A5 and ZFHX4 among the LUAD patients (Table S4). The expression level of each protein was detected by IHC analysis (Fig. 3d). As shown in Fig. 3e, f, high expression of the TWIST1-ceRNET components was significantly correlated with lymph node metastasis and advanced TNM staging (Lymph node metastasis: *P* = 0.025 for TWIST1, *P* = 0.005 for SLC12A5, *P* < 0.001 for ZFHX4; TNM stage: *P* = 0.008 for TWIST1, *P* = 0.015 for SLC12A5, *P* = 0.012 for ZFHX4;). Overall survival (OS) was calculated by Kaplan–Meier analysis and the log-rank test. As shown in Fig. 3g–i, patients with higher TWIST1-ceRNET expression had poor OS (TWIST1, *P* = 0.003; SLC12A5, *P* = 0.021; ZFHX4, *P* = 0.022).

### Biological functions of the TWIST1 ceRNET in vitro and in vivo

Prior to conducting the biological study of TWIST1 ceRNET in LUAD in vitro, we first evaluated the expression profile of TWIST1 in LUAD cancer cell lines by RT-PCR analysis. TWIST1 was relatively highly expressed in the H1299 cell line, but lowly expressed in the A549 cell line (Supplementary Fig. 1). Accordingly, we selected these two cell lines for the following study.

**Fig. 4:**
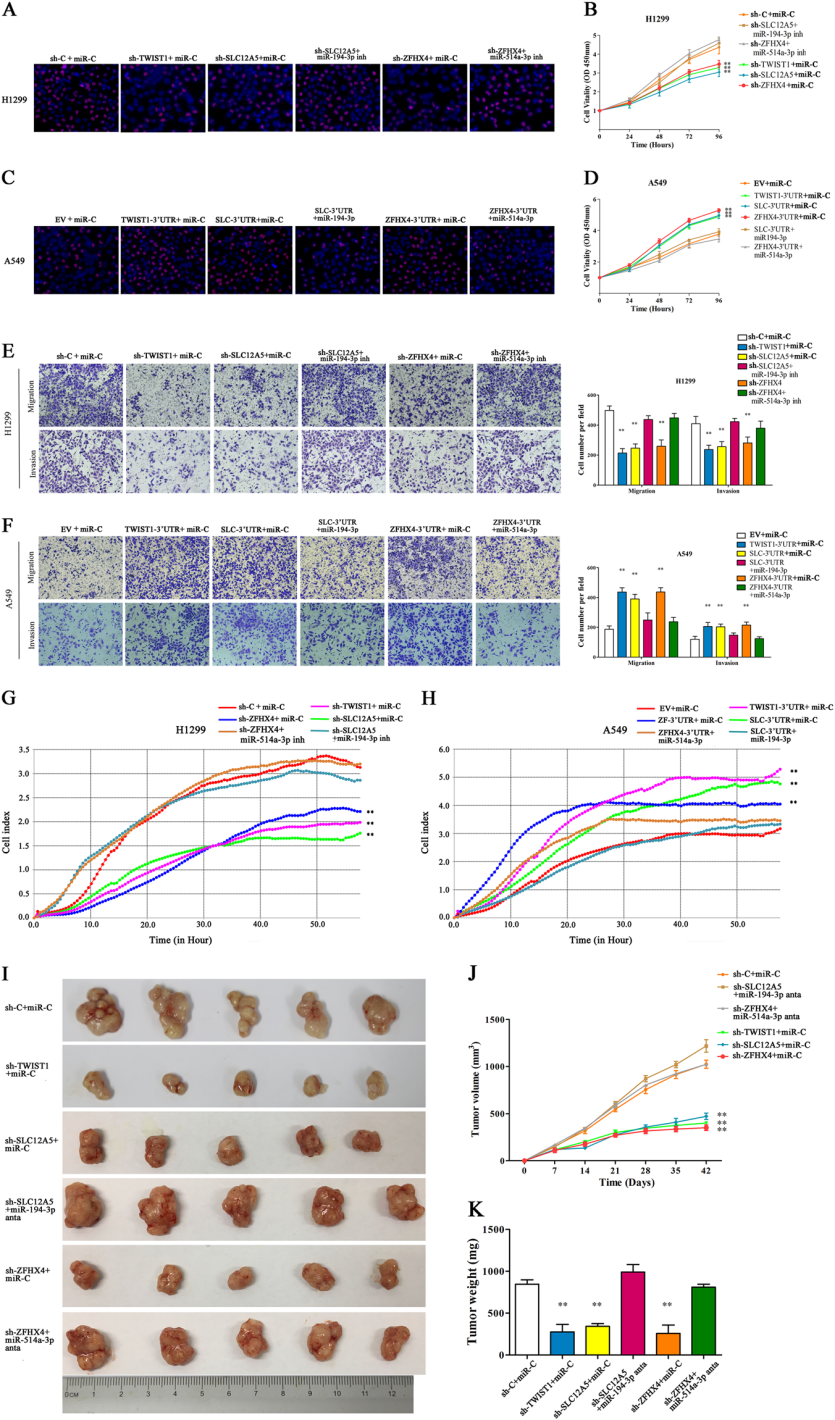
Biological functions of the TWIST1 ceRNET in vitro and in vivo. **a**–**d** Effects of TWIST1-ceRNET components on proliferative capacity of H1299 (**a**, **b**) cells and A549 (**c**, **d**) cells evaluated by 5-ethynyl-2′-deoxyuridine (EdU) proliferation assay (**a**, **c**) and Cell Counting Kit-8 (CCK-8) assay (**b**–**d**). **e**–**h** Effects of TWIST1-ceRNET components on migration and invasion capacity of H1299 (**e**, **g**) cells and A549 (**f**, **h**) cells evaluated by transwell assay, matrigel assay (**e**, **f**) and xCELLigence System assay (**g**, **h**). **i**–**k** The effects of TWIST1-ceRNET components on tumor growth in Xenograft tumor models. In vivo tumor lumps (**I**, *n* = 5) of xenograft mouse models composed of H1299 cells, which were treated with miR-194-3p or miR-514a-3p antagomirs as well as sh-TWIST1/sh-SLC12A5/sh-ZFHX4. The tumor growth curves (**j**) and the mean tumor weight (**k**) of each group. All data are mean ± s.d. The data statistical significances were assessed by Student’s *t*-test compared to the NC group. *P < 0.05, **P < 0.001

To investigate the biological function of the TWIST1-centered ceRNET, we designed shRNAs targeting TWIST1/SLC12A5/ZFHX4. Specific 3′UTR regions containing the predicted binding sites were also designed and inserted into the expression vector pcDNA3.1. Overexpression of specific 3′UTR regions were confirmed by RT-PCR using primers targeting the 3′UTR regions (Supplementary Fig. 2). By evaluating cell proliferation using the EdU proliferation assay and the CCK-8 assay, we found that knockdown of TWIST1/SLC12A5/ZFHX4 led to a significant decrease in the proliferative capacity of H1299 cells (Fig. 4a, b), whereas overexpression of the TWIST1/SLC12A5/ZFHX4 3′UTRs significantly increased the proliferative capacity of A549 cells (Fig. 4c, d). The upregulatory or downregulatory effect caused by SLC12A5 and ZFHX4 could be eliminated when a mimics/inhibitor of miR-194-3p or miR-514a-3p was transfected into the cells (Fig. 4a–d). The effects of TWIST1 ceRNET on the migration and invasion of H1299/A549 cells were also determined using the transwell assay, matrigel assay and xCELLigence System assay. These effects were also eliminated by transfection of mimics/inhibitor of miR-194-3p and miR-514a-3p (Fig. 4e–h). Thus, the results of the in vitro experiments suggested that the TWIST1-ceRNET promotes the proliferation, migration and invasion of LUAD cells.

Then we tested the phenotypic consequence of over-expressing the cDNAs (devoid of 3′UTRs) of TWIST1/SLC12A5/ZFHX4 in A549 cell line, along with the miRNA mimics. As a result, overexpression of the TWIST1/SLC12A5/ZFHX4 cDNAs significantly increased the proliferative, invasion and migration capacity of A549 cells (Supplementary Fig. 3). The upregulatory effect caused by SLC12A5 and ZFHX4 could not be eliminated when a mimics/inhibitor of miR-194-3p or miR-514a-3p was transfected into the cells.

Xenograft tumor models were used to assess the oncogenic role of TWIST1-ceRNET in vivo. We used six groups similar to the in vitro experiments (Fig. 4i). MicroRNA antagomirs exert anti-microRNA function as microRNA inhibitors, but the effect lasts longer and is steadier. Compared with the control group, the tumor volume and tumor weight were smaller in the sh-TWIST1/sh-SLC12A5/sh-ZFHX4 groups. However, the growth trend due to sh-SLC12A5 and sh-ZFHX4 was reversed by miR-194-3p and miR-514a-3p antagomirs, respectively (Fig. 4i–k).

### TWIST1 ceRNAs regulate TWIST1 expression in LUAD cell lines

**Fig. 5:**
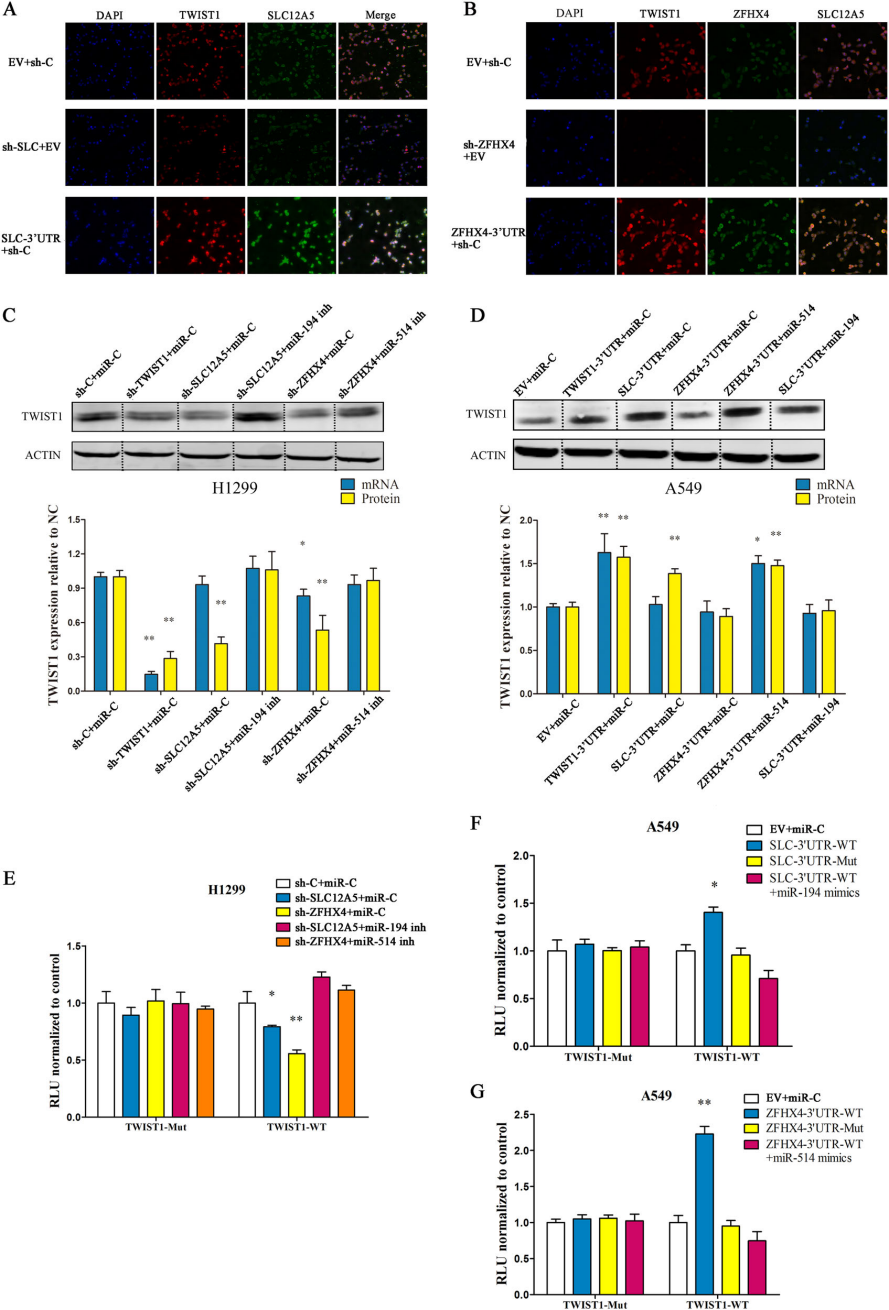
TWIST1 ceRNAs regulate TWIST1 expression in LUAD cell lines. **a**, **b** Double-label staining showed that TWIST1 expression was significantly increased after transfection with SLC12A5-3′UTR (**a**)/ZFHX4-3′UTR (**b**), and decreased after transfection with sh-SLC12A5/ZFHX4 in H1299 cells. **c** Western blot for TWIST1 protein levels (upper) and quantitation of TWIST1 protein and mRNA levels (lower) in H1299 cells transfected with miR-194-3p or miR-514a-3p inhibitors as well as sh-TWIST1/sh-SLC12A5/sh-ZFHX4. **d** Western blot for TWIST1 protein levels (upper) and quantitation of TWIST1 protein and mRNA levels (lower) in A549 cells transfected with miR-194-3p or miR-514a-3p mimics as well as TWIST1-3′UTR/ SLC12A5-3′UTR / ZFHX4-3′UTR regions. **e** Luciferase activity in H1299 cells co-transfected with shRNA against TWIST1 ceRNAs as well as miR-194-3p or miR-514a-3p inhibitors and a luciferase-TWIST1 3′UTR reporter construct. **f**, **g** Luciferase activity in H1299 cells co-transfected with 3′UTR regions of SLC12A5 (**f**) and ZFHX4 (**g**) as well as miR-194-3p or miR-514a-3p mimics and a luciferase-TWIST1 3′UTR reporter construct. All data are mean ± s.d. The data statistical significances were assessed by Student’s t-test compared to the NC group. *P < 0.05, **P < 0.001

We then investigated the ability of SLC12A5/ZFHX4 to regulate TWIST1 expression in LUAD cell lines. First, we performed double-label staining of TWIST1 + SLC12A5 and TWIST1 + ZFHX4 to evaluate the regulatory effects of SLC12A5/ZFHX4 on TWIST1 expression in H1299 cells. As shown in Fig. 5a, b, TWIST1 expression was significantly increased after transfection with SLC12A5-3′UTR/ZFHX4-3′UTR, and decreased after transfection with sh-SLC12A5/ZFHX4. Western blot analysis and RT-PCR analysis were also performed to examine the effects of SLC12A5/ZFHX4 on TWIST1 expression in LUAD cells. In H1299 cells, knock-down of SLC12A5/ZFHX4 suppressed the expression of TWIST1 at both the mRNA and protein level, which was reversed by inhibition of miR-194-3p and miR-514a-3p, respectively (Fig. 5c). In A549 cells, transfection of SLC12A5/ZFHX4 3′UTR upregulated the expression of TWIST1, which was reversed by miR-194-3p and miR-514a-3p mimics (Fig. 5d).

In addition, we investigated the effect of the expression of the TWIST1 3′UTR region in LUAD cells by transfecting the luciferase reporter plasmid psiCHECK-2 carrying wild-type TWIST1 3′UTR region or mutant TWIST1 3′UTR region into LUAD cells. For instance, in H1299 cells, knock-down of SLC12A5/ZFHX4 decreased the luciferase activity of the wild-type group, but this effect was abolished by the miR-194-3p inhibitor and miR-514a-3p inhibitor, respectively (Fig. 5e). Similarly, in A549 cells, transfection of the wild-type SLC12A5/ZFHX4 3′UTR regions decreased the luciferase activity of the TWIST1 wild-type group, but the effect was also abolished by the miR-194-3p mimics and miR-514a-3p mimics, respectively (Fig. 5f, g). These results indicated that the 3′UTR regions of SLC12A5/ZFHX4 can regulate TWIST1 expression by targeting the TWIST 3′UTR region in LUAD cell lines.

### miR-194-3p and miR-514a-3p regulate the TWIST1-ceRNET

**Fig. 6:**
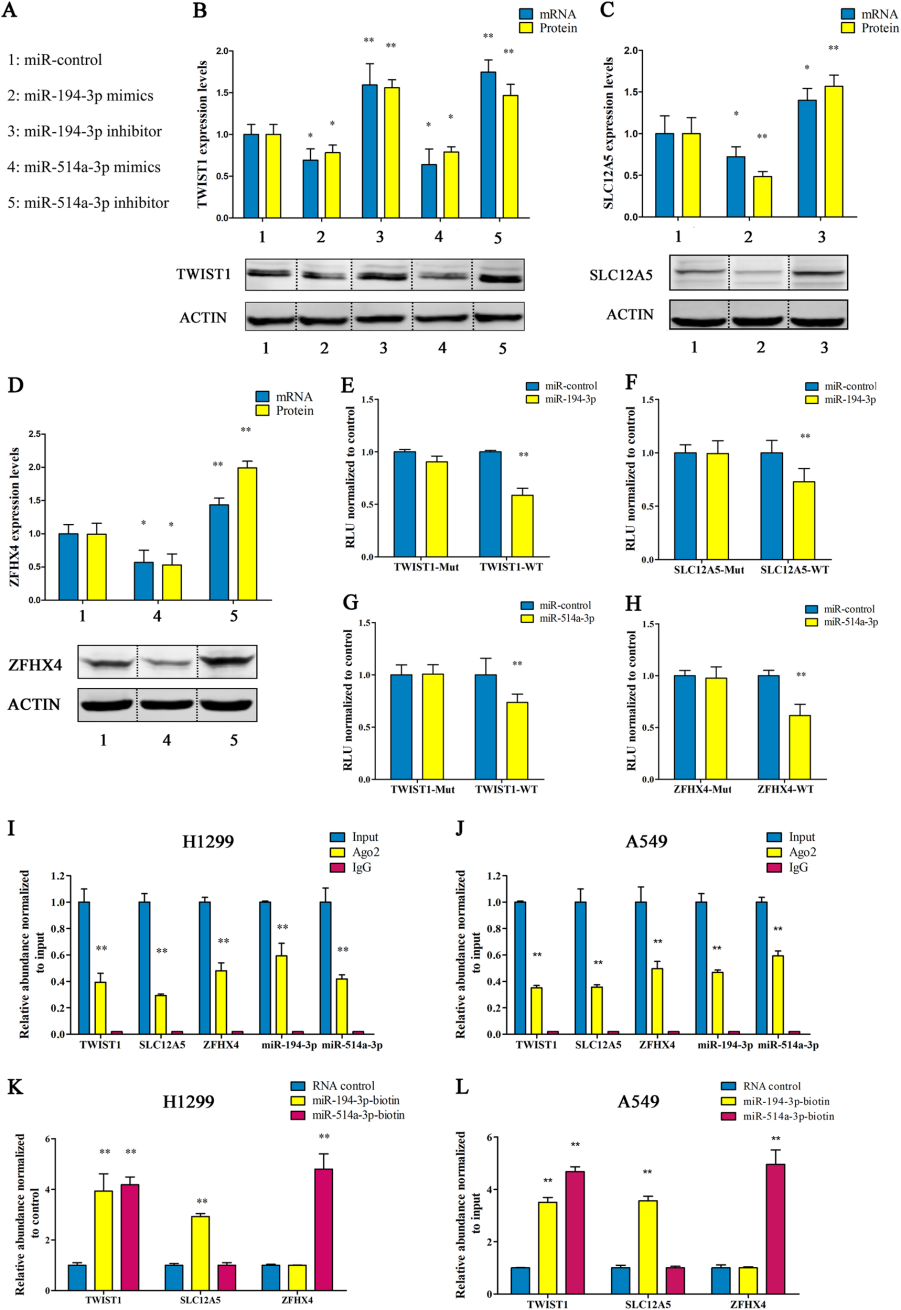
miR-194-3p and miR-514a-3p regulate the TWIST1-ceRNET. **a** Schematic drawing of the groups design. **b** Western blot for TWIST1 protein levels (lower) and quantitation of TWIST1 protein and mRNA levels (upper) in H1299 cells after knockdown or overexpression of miR-194-3p or miR-514a-3p. **c** Western blot for SLC12A5 protein levels (lower) and quantitation of SLC12A5 protein and mRNA levels (upper) in H1299 cells after knockdown or overexpression of miR-194-3p. **d** Western blot for ZFXH4 protein levels (lower) and quantitation of ZFXH4 protein and mRNA levels (upper) in H1299 cells after knockdown or overexpression of miR-514a-3p. **e**, **f** Luciferase activity in H1299 cells co-transfected with miR-194-3p mimics and a luciferase-TWIST1 3′UTR (**e**) or luciferase-SLC12A5 (**f**) reporter construct. **g**, **h** Luciferase activity in H1299 cells co-transfected with miR-514a-3p mimics and a luciferase-TWIST1 3′UTR (**g**) or luciferase-ZFHX4 (**h**) reporter construct. **i**, **j** TWIST1/SLC12A5/ZFHX4, together with miR-194-3p and miR-514a-3p were all efficiently pulled down by anti-Ago2 in H1299 (I) or A549 (**j**) cells. **k**, **l** TWIST1 and SLC12A5 were efficiently enriched by biotin-coupled miR194-3p mimics, while TWIST1 and ZFHX4 were efficiently enriched by biotin-coupled miR-514a-3p mimics in H1299 (**k**) or A549 (**l**) cells. All data are mean ± s.d. The data statistical significances were assessed by Student’s t-test compared to the NC group. *P < 0.05, **P < 0.001

Previous study indicated that miR-194-3p and miR-514a-3p play an important role in the regulation of the TWIST1-ceRNET. We investigated the ability of miR-194-3p/miR-514a-3p to regulate the TWIST1-ceRNET by first examining the effect of overexpression or knock-down of these two microRNAs on the expression of levels of the TWIST1-ceRNET components in H1299 cells. Five groups were used to simplify the study (Fig. 6a). In general, knock-down of miR-194-3p resulted in a significant increase in the expression level of the TWIST1/SLC12A5 proteins and mRNAs, while overexpression of miR-194-3p significantly reduced the expression level of the TWIST1/SLC12A5 proteins and mRNAs (Fig. 6b, c). Similarly, overexpression of miR-514a-3p reversely regulated the expression level of TWIST1/ZFHX4 proteins and mRNAs (Fig. 6b, d). We also investigated the effect of the expression of the TWIST1/SLC12A5/ZFHX4 3′UTR regions by transfecting luciferase reporter plasmids psiCHECK-2 carrying the wild-type or mutant TWIST1/SLC12A5/ZFHX4 3′UTR regions into H1299 cells. The results revealed that overexpression of wild-type miR-194-3p and miR-514a-3p, but not miR-mutant or miR-NC, decreased the luciferase activity of the plasmids carrying the wild-type 3′UTR regions (Fig. 6e–h). As for the ceRNA mechanism, it is possible, based on previous studies ^28–30^, that the prevalent phenomenon is that AGO2 binds with mRNAs and miRNAs^39,41,42^. We therefore conducted a RIP assay to pull down RNA transcripts that bind to AGO2 in H1299 and A549 cells. Eventually, TWIST1/SLC12A5/ZFHX4, together with miR-194-3p and miR-514a-3p were all efficiently pulled down by anti-Ago2 (Fig. 6i, j). Moreover, we overexpressed 3′UTR regions of SLC12A5 and ZFHX4 then pulled down Ago2. Overexpression of 3′UTR regions of SLC12A5 and ZFHX4 both caused a significant decrease in the enrichment of TWIST1 transcripts pulled down by Ago2 (Supplementary Figs. 4, 5), indicating that there were less miRNA-bound TWIST1 transcripts. This suggests that SLC12A5 and ZFHX4 can compete with the TWIST1 transcript for the binding of miRNAs. To further evaluate whether the 3′UTR regions of this ceRNET could sponge miRNAs, we performed a miRNA pull-down assay using biotin-coupled miR-194-3p and miR-514a-3p mimics. We found that TWIST1 and SLC12A5 were efficiently enriched by miR194-3p, while TWIST1 and ZFHX4 were efficiently enriched by miR-514a-3p (Fig. 6k, l).

## Discussion

An increasing number of studies have focused on the ceRNA mechanism in LUAD progression, these studies to some extent proved that the ceRNA language expands the total collection of functional genetic information in our genome which may play a key role in tumor progression. Notably, interference of gene expression can be potentially transmitted in the ceRNET through a cascade of co-regulated target RNAs and microRNAs that share targets, leading to mutual effects between distant components in the network. As mentioned above, mRNAs should exert the most influence on the ceRNA language and make up the more complex and powerful ceRNET. However, to date, more attention has been paid to the role of non-coding RNAs in ceRNA crosstalk.

In the present study, we first constructed ceRNA interactomes based on the 5744 miRNA-mRNA pairs predicted by TargetScan, miRanda and Starbase. Then, the expression data obtained from TCGA database were analyzed to screen out the co-expression ceRNA pairs. Finally, 34,706 pairs of mRNAs were screened out and create the ceRNET for LUAD. This network provides important clues for exploring the key mRNAs and associated regulatory network in the pathogenesis of LUAD. With the help of the ceRNET, we located the TWIST1-centered ceRNET, which recruits SLC12A5 and ZFHX4 as its ceRNAs.

TWIST1 has been shown to be associated with tumor recurrence, metastasis and poor prognosis in different types of human cancers^43–47^. In the present study, we confirmed that high TWIST1 expression promoted proliferation and metastasis of LUAD cell lines and LUAD patients with high TWIST1 expression had a shorter OS. However, the ceRNA network indicated that TWIST1 could also function as a hub gene of ceRNET, which has not been reported until now. Data from TCGA database, together with the RT-PCR analysis of 38 fresh LUAD tissue samples and IHC staining of 42 paraffin-embedded tissue samples suggested that SLC12A5 and ZFHX4 were co-expressed with TWIST1. As potential ceRNAs of TWIST1, the function of SLC12A5 and ZFHX4 has not been adequately studied in LUAD. Our study showed that the expression level of SLC12A5 and ZFHX4 was higher in LUAD tissue samples than in normal lung tissue samples. Moreover, high expression of SLC12A5 and ZFHX4 was associated with more aggressive LUAD characteristics and poor prognosis.

We further confirmed the oncogenic effects of TWIST1-centered ceRNET in vitro and in vivo. The effects of SLC12A5 and ZFHX4 were reversed by miR-194-3p and miR-514a-3p, indicating that SLC12A5 and ZFHX4 exerted their effects by regulating TWIST1 expression. Our study also determined that TWIST1/SLC12A5/ZFHX4 can function as a ceRNET by competitively binding with miR-194-3p and miR-514a-3p. As TWIST1 ceRNAs, SLC12A5 and ZFHX4 influenced the mRNA and protein expressions of TWIST1 in 3′UTR- and miRNA-dependent manners, which were also corroborated by the luciferase reporter assay results. In addition, miR-194-3p and miR-514a-3p were found to modulate the expression of TWIST1, SLC12A5 and ZFHX4. The results of the AGO2-RIP assay revealed evidence about the likely TWIST1 ceRNA regulatory mechanism. Additionally, the results of the luciferase reporter assay and biotin pull-down assay confirmed that miR-194-3p and miR-514a-3p can directly target the 3′UTR regions of TWIST1, SLC12A5, and ZFHX4, thus forming a TWIST1-centered ceRNET.

The proposed ceRNA mechanism provocatively challenges the traditional definition of the mRNA function^48,49^. As for our research, the well-known critical EMT-inducing transcription factor TWIST1 was identified an unexpected dimension whereby RNA molecules can communicate and exert their functions. Additionally, with the help of the ceRNA network we constructed in LUAD, two potential oncogenic genes were predicted and proved to be involved in the TWIST1-centered ceRNET by targeting miR-194-3p and miR-514a-3p. Our study describes the ceRNET of SLC12A5 and SLC12A5 can function as components of a sub-ceRNET by competitively binding with miR-194-3p/miR-514a-3p, and therefore upregulated the expression of TWIST1, thus promoted the proliferation, invasion, and migration of LUAD.

We can reach some meaningful conclusions from the findings we presented. According to our results, the crosstalk between mRNA transcripts endowed themselves new function in addition to their well-proved functions. It’s also established that the trans modulator role of a given mRNA can be predicted through the ceRNA network based on bioinformatic methods. We believe that by the method we supplied in this research, the potential ceRNA function of more known or unknown oncogenic mRNAs would be found to complete the carcinogenesis profile.

## Conclusion

To sum up, we constructed the mRNA-mRNA related ceRNET for LUAD and found that SLC12A5 and ZFHX4 exert their oncogenic function by regulating TWIST1 expression through a ceRNA mechanism.

## Supplementary information

supplementary materials

## References

[1] Bray Freddie, Ferlay Jacques, Soerjomataram Isabelle, Siegel Rebecca L., Torre Lindsey A., Jemal Ahmedin (2018). Global cancer statistics 2018: GLOBOCAN estimates of incidence and mortality worldwide for 36 cancers in 185 countries. CA: A Cancer Journal for Clinicians.

[2] Salmena L, Poliseno L, Tay Y, Kats L, Pandolfi PP (2011). A ceRNA hypothesis: the Rosetta Stone of a hidden RNA language?. Cell.

[3] Cazalla D, Yario T, Steitz JA (2010). Down-regulation of a host microRNA by a Herpesvirus saimiri noncoding RNA. Science.

[4] Ebert MS, Neilson JR, Sharp PA (2007). MicroRNA sponges: competitive inhibitors of small RNAs in mammalian cells. Nat. Methods.

[5] Esposito F (2014). HMGA1 pseudogenes as candidate proto-oncogenic competitive endogenous RNAs. Oncotarget.

[6] Franco-Zorrilla JM (2007). Target mimicry provides a new mechanism for regulation of microRNA activity. Nat. Genet..

[7] Karreth FA (2015). The BRAF pseudogene functions as a competitive endogenous RNA and induces lymphoma in vivo. Cell.

[8] Tay Y (2011). Coding-independent regulation of the tumor suppressor PTEN by competing endogenous mRNAs. Cell.

[9] Tay Y, Karreth FA, Pandolfi PP (2014). Aberrant ceRNA activity drives lung cancer. Cell Res..

[10] Wang H (2017). STAT3-mediated upregulation of lncRNA HOXD-AS1 as a ceRNA facilitates liver cancer metastasis by regulating SOX4. Mol. Cancer.

[11] Yang XZ (2018). LINC01133 as ceRNA inhibits gastric cancer progression by sponging miR-106a-3p to regulate APC expression and the Wnt/beta-catenin pathway. Mol. Cancer.

[12] Zhang G (2018). LncRNA MT1JP functions as a ceRNA in regulating FBXW7 through competitively binding to miR-92a-3p in gastric cancer. Mol. Cancer.

[13] Sui J (2017). Integrated analysis of competing endogenous RNA network revealing lncRNAs as potential prognostic biomarkers in human lung squamous cell carcinoma. Oncotarget.

[14] Bartel DP (2009). MicroRNAs: target recognition and regulatory functions. Cell.

[15] Cabili MN (2011). Integrative annotation of human large intergenic noncoding RNAs reveals global properties and specific subclasses. Genes Dev..

[16] Chi SW, Zang JB, Mele A, Darnell RB (2009). Argonaute HITS-CLIP decodes microRNA-mRNA interaction maps. Nature.

[17] Derrien T (2012). The GENCODE v7 catalog of human long noncoding RNAs: analysis of their gene structure, evolution, and expression. Genome Res..

[18] Grither WR (2018). TWIST1 induces expression of discoidin domain receptor 2 to promote ovarian cancer metastasis. Oncogene.

[19] Lee KW, Yeo SY, Sung CO, Kim SH (2015). Twist1 is a key regulator of cancer-associated fibroblasts. Cancer Res..

[20] Malek R (2017). TWIST1-WDR5-Hottip regulates Hoxa9 chromatin to facilitate prostate cancer metastasis. Cancer Res..

[21] Schirosi L (2017). VEGF and TWIST1 in a 16-biomarker immunoprofile useful for prognosis of breast cancer patients. Int. J. Cancer.

[22] Brabletz T (2012). EMT and MET in metastasis: where are the cancer stem cells?. Cancer Cell.

[23] Eide T, Ramberg H, Glackin C, Tindall D, Tasken KA (2013). TWIST1, A novel androgen-regulated gene, is a target for NKX3-1 in prostate cancer cells. Cancer Cell Int..

[24] Hung JJ (2009). Prognostic significance of hypoxia-inducible factor-1alpha, TWIST1 and Snail expression in resectable non-small cell lung cancer. Thorax.

[25] Qin Q, Xu Y, He T, Qin C, Xu J (2012). Normal and disease-related biological functions of Twist1 and underlying molecular mechanisms. Cell Res..

[26] Hebert SC, Mount DB, Gamba G (2004). Molecular physiology of cation-coupled Cl- cotransport: the SLC12 family. Pflug. Arch.: Eur. J. Physiol..

[27] Payne JA, Stevenson TJ, Donaldson LF (1996). Molecular characterization of a putative K-Cl cotransporter in rat brain. A neuronal-specific isoform. J. Biol. Chem..

[28] Song L (2002). Molecular, functional, and genomic characterization of human KCC2, the neuronal K-Cl cotransporter. Brain Res. Mol. Brain Res..

[29] Liu JY (2017). Solute carrier family 12 member 5 promotes tumor invasion/metastasis of bladder urothelial carcinoma by enhancing NF-kappaB/MMP-7 signaling pathway. Cell Death Dis..

[30] Xu L (2016). Increased expression of Solute carrier family 12 member 5 via gene amplification contributes to tumour progression and metastasis and associates with poor survival in colorectal cancer. Gut.

[31] Yu C (2014). Discovery of biclonal origin and a novel oncogene SLC12A5 in colon cancer by single-cell sequencing. Cell Res..

[32] Yu J (2015). Novel recurrently mutated genes and a prognostic mutation signature in colorectal cancer. Gut.

[33] Kostich WA, Sanes JR (1995). Expression of zfh-4, a new member of the zinc finger-homeodomain family, in developing brain and muscle. Developmental Dyn..

[34] Chudnovsky Y (2014). ZFHX4 interacts with the NuRD core member CHD4 and regulates the glioblastoma tumor-initiating cell state. Cell Rep..

[35] Betel D, Koppal A, Agius P, Sander C, Leslie C (2010). Comprehensive modeling of microRNA targets predicts functional non-conserved and non-canonical sites. Genome Biol..

[36] Lewis BP, Burge CB, Bartel DP (2005). Conserved seed pairing, often flanked by adenosines, indicates that thousands of human genes are microRNA targets. Cell.

[37] Li JH, Liu S, Zhou H, Qu LH, Yang JH (2014). starBasev2.0: decoding miRNA-ceRNA, miRNA-ncRNA and protein-RNA interaction networks from large-scale CLIP-Seq data. Nucleic Acids Res..

[38] Yang X (2013). Glypican-5 is a novel metastasis suppressor gene in non-small cell lung cancer. Cancer Lett..

[39] Zheng Q (2016). Circular RNA profiling reveals an abundant circHIPK3 that regulates cell growth by sponging multiple miRNAs. Nat. Commun..

[40] Shannon P (2003). Cytoscape: a software environment for integrated models of biomolecular interaction networks. Genome Res..

[41] Wang K (2017). Circular RNA mediates cardiomyocyte death via miRNA-dependent upregulation of MTP18 expression. Cell Death Differ..

[42] Yu CY (2017). The circular RNA circBIRC6 participates in the molecular circuitry controlling human pluripotency. Nat. Commun..

[43] Elloul S (2005). Snail, Slug, and Smad-interacting protein 1 as novel parameters of disease aggressiveness in metastatic ovarian and breast carcinoma. Cancer.

[44] Mironchik Y (2005). Twist overexpression induces in vivo angiogenesis and correlates with chromosomal instability in breast cancer. Cancer Res..

[45] Moody SE (2005). The transcriptional repressor Snail promotes mammary tumor recurrence. Cancer Cell.

[46] Yang J (2004). Twist, a master regulator of morphogenesis, plays an essential role in tumor metastasis. Cell.

[47] Yang MH, Wu KJ (2008). TWIST activation by hypoxia inducible factor-1 (HIF-1): implications in metastasis and development. Cell Cycle.

[48] de Giorgio A, Krell J, Harding V, Stebbing J, Castellano L (2013). Emerging roles of competing endogenous RNAs in cancer: insights from the regulation of PTEN. Mol. Cell. Biol..

[49] Su X (2013). microRNAs and ceRNAs: RNA networks in pathogenesis of cancer. Chin. J. Cancer Res. = Chung-kuo yen cheng yen chiu.

