# Supplementary material for: The TWIST1-centered competing endogenous RNA network promotes proliferation, invasion, and migration of lung adenocarcinoma
Source: Oncogenesis. 2019 Oct 23;8(11):62. doi: 10.1038/s41389-019-0167-6 (PMC6811597; doi:10.1038/s41389-019-0167-6)

Supplementary table

Table S1. The primers of shRNA.

Gene	Primer (5'→3')
Sh-TWIST1	TCGAGGTGTCTAAATGCATTCATATTCAAGAGAAATATGAATGCATTTAGACACTTTTTGC
Sh-SLC12A5	GATCGGCAGCACAACACTGTGCTTGTTTCAAGAGAACAAGCACAGTGTTGTGCTGCTTTTTTC
Sh-ZFHX4	CCGGCGGACAGTTCTTGCCATACTTCTCGAG-AAGTATGGCAAGAACTGTCCGTTTTT

Table S2. Sequence synthesized for the luciferase reporter assay

Purpose	Primer（5'→3'）
ZFHX4 UTR-pcDNA3.1(+)-WT	UAACAUUUUUGUUUUGCUUGCCAUGAAUUUCAACUUCCACCACCCAGUGAAUUGAUUUAUAAAUUGCUAUGCUUUGCUGUUUUUCUGUUGCUGUGGAACUUAAAGAAUGUGAAAGCUGUCAAAGGGUAUUUUACGAAUCACUUUUGUGUUUGAUAUAGUAAAACAAUGUGAUUCAUUCCAAAGUAACAGAAGGUUAUUUGUAAGAAAGUUAAAGGCUUGUGAACAAAGAAAGCUAAG
ZFHX4 UTR-psiCHECK-2-WT	UAACAUUUUUGUUUUGCUUGCCAUGAAUUUCAACUUCCACCACCCAGUGAAUUGAUUUAUAAAUUGCUAUGCUUUGCUGUUUUUCUGUUGCUGUGGAACUUAAAGAAUGUGAAAGCUGUCAAAGGGUAUUUUACGAAUCACUUUUGUGUUUGAUAUAGUAAAACAAUGUGAUUCAUUCCAAAGUAACAGAAGGUUAUUUGUAAGAAAGUUAAAGGCUUGUGAACAAAGAAAGCUAAG
TWIST1-514a-3p- psiCHECK-2-WT	UCAUGGGGAGUCUCCUUGUUCACUAGCUUGGUCUGACUAUUUGAUAUUUCAGAUUUCUUAAGGUACACCAGGAUGGAGCUGGCCAUGACUGCUGAGAAGGUAAUUGUUAUCUUAACUCUGGAUAUAAGAACUAGAUGUGUCAAAGAAAGGUAGCUCUCCAUUUGCCUUUCUUUUAUUUCUUUCUUUUUUUAAUGUGUUUUGCUUUUUCUACUUUUUUCAUAAGGGGAAACACUUUGGA
TWIST1-194-3p-psiCHECK-2-WT	UCUGCAUUCUGAUAGAAGUCUGAACAGUUGUUUGUGUUUUUUUUUUUUUUUUUUUUGACGAAGAAUGUUUUUAUUUUUAUUUUUUUCAUGCAUGCAUUCUCAAGAGGUCGUGCCAAUCAGCCACUGAAAGGAAAGGCAUCACUAUGGACUUUCUCUAUUUUAAAAUGGUAACAAUCAGAGGAACUAUAAGAACACCUUUAGAAAUAAAAAUACUGGGAUCAAACUGGCCUGCAAAACCAU
TWIST1-UTR-pcDNA3.1(+)-WT	GUCGUGCCAAUCAGCCACUGAAAGGAAAGGCAUCACUAUGGACUUUCUCUAUUUUAAAAUGGUAACAAUCAGAGGAACUAUAAGAACACCUUUAGAAAUAAAAAUACUGGGAUCAAACUGGCCUGCAAAACCAUAGUC

	AGUUAAUUCUUUUUUUCAUCCUUCCUCUGAGGGGAAAACAAAAAA<br>AAACUUAAAAUACAAAAAACAACAUUCUAUUUAUUUAUUGAGGACC<br>CAUGGUAAAAUGCAAAUAGAUCCGGUGUCUAAAUGCAUUCAUAUUU<br>UUAUGAUUGUUUUGUAAAUAUCUUUGUAUAUUUUCUGCAAUAAAU<br>AAAUAUAAAAAAUUUAGAGAACCUUAGAGUUUGGUCUAUAUUUUUA<br>AAACUAAAGAUUAAGUUGGUGGUAAAUACCUGCUUGUUUAAUUCUA<br>GAGGCACCCAGGAGGGAGGGGGCACUAAUAUAAACAAAGCAAUGAA<br>AAACUCAAAUAAAGCAGCUACUGACAGGCACAAGCAUGUUAUUUUA<br>AAAGACAGCUUUAUUAUUAUUCCAGUUUGGUAUUCAGAGGGCUUAG<br>UAGCAUCUCUUCAUCUCUUACUGUCUCCAAACAGCAAAAACUUAAC<br>AAAUUUGCUAUUCACCUCCUUUUUAUUUUGAGGUGAUAGUUGUUAA<br>AACAGUUAAAUGCAAAUAAGGAGUAUAAAACCUGCACUAUUCUCUU<br>AUUAUGUACUUUUUGUCAUGCUCUUCACUCUUUGUGAAGAUUCCUA<br>AAUAACAGGUUCUUUUCAGUGAAAACAUAUAUAGUGUUAUGAAAAU<br>UAUCUAUACACAUAUAGAAAUGACCAGAAAUGCAUAGAAAAUAAAG<br>UGUAAAUCAGCAGAAAAGAGAAAAUUCCAUUUGUAAUUUGCUUAAC<br>UCUUCUCUCAUGAAUGAAUAUGACGUUUAGUGUCAUGUUUGCUAAA<br>AAGAAAAGUUAAUAUAUCGCUAUAUUGACGCUAUUUGGGAAGCAUC<br>AUUCUUUUUUUAUGUGUAAAGAAUAUAAGGCUGUGGAAUAAUAUAC<br>AAAAAUGAAGAUCCUGAACUUUCAGUAUCCUUGGUAACUGGAUAUA<br>AUCACAGUUGGAAUAAUCACAGUUUUAAAUACUACAGCUAUGCAGU<br>UACUAGUCAGUUCAUUAGCGUUAUAUUUUAUCCAGAAUUACACAGA<br>AUUUUUCCCUGGUGAGAUCAUACAUACACACACACACAAUCUGAAG<br>UUAAGUAAAACAAUAAUGGGCAAACGGGGGUGUUUGAUUUUUUAUU<br>UCAUUCUGAUUAUUUGUGGGCCAGGAACAUACUGGCUUUUAUAAUU<br>AACAUUAUUAGAAAUAGUGAUUUUGUAUCAGUUCUUAGCUUUUACA<br>UCCGAUUUACUGCAUCCAAGUGUAACUGGGGAAGCUUUCAAACUGG<br>AAAAUCAUUUUGGUGCCUGUUUCAAACAAUGACUAAUAAGUGUAUG<br>UAUCCAAACAGAUCGUUUUUGCUUAGCUCAGGUCAAAGUUAUUUUA<br>UUGCCUGAAGGUUUUUGUUUUCUUCUUCACACAGCACCAUAGAAUG<br>CUGACAUAAGAAUAUUAUGAAUUUAUUGUUAAUAUUUCAGACUAUA<br>UUUUACAAUAAGGAGUCUGCAGCAUUGACUUGUAUUAAAAGGUUAG<br>UGAAAGCUUGGAGAAGUAUUAUUAUUUUUUUAUUUUGAUAGUCCUU<br>UAGCAAAUAACAGAAAGGGAAGGCGAUUAUGUGUUGGUGUAUUUGU<br>UGGGGGUGUGUGUGUGUUGUGUGUAAGGCAGGUUAGAAAUGUAAGG<br>AGGGUGCCCUAGGAGGAUGAGGGAAAGCAUUAGGUUGGUAAUUCUU<br>UUUUGUAUGUAUCAUCCAACCACCUCCAUUUUCUAAAUUAUUCAUG<br>GGGAGUCUCCUUGUUCACUAGCUUGGUCUGACUAUUUGAUAUUUCA<br>GAUUUCUUAAGGUACACCAGGAUGGAGCUGGCCAUGACUGCUGAGA<br>AGGUAAUUGUUAUCUUAACUCUGGAUAUAAGAACUAGAUGUGUCAA<br>AGAAAGGUAGCUCUCCAUUUGCCUUUCUUUUAUUUCUUUCUUUUUU<br>UAAUGUGUUUUGCUUUUUCUACUUUUUUCAUAAGGGGA
SLC12A5 UTR-<br>pcDNA3.1(+)-	UCGCUGGGGAGCAGGCGUCUCUCCUCAGUCGGCUUGUCGCCUGCUCC<br>CCGUAUCCCAUGGCUCCUCGCCAAAGACUGAAAUUGUGGAGCUGGA

WT	GGGCGCCCCCUCCCCGGAGUUUCCUCCCUGGGACAAGUGAGGGAGGA<br>GGGGGCCGAUUCUGGUUUAGGGGCCGGACCCACUGAGAGGCCCCAG<br>AGCCGCCCGUGAUGUUCCUCCCCCGUCCCCAUCUGGCAGCUCCUGUC<br>UCGCCUGAGGGACCCAGCCGCCUUCUCCGUGCUCUGGGGCCGGGCCU<br>CGCUGCUUAGCAGCGGCCUCUAGCUCCGUCUCCCGGGGACCUGGGCC<br>UGAGGGAGGGCUGGAGUCGCACGCGCUUUGUCCUUAGCGCCUGUCU<br>GCUCUCCUCUAACUAGGACCCAGGGCCUUUGGCUUCCCCAGCUCAUC<br>CUUGGCCCUUCCGCUCCACCAGCCUGGUCUGAGGCGUGCUCUGUCCU<br>UAGAGAAGGCGCGGUGGCCGGGUUCCCUUCCCCUAGGGCACAUUAC<br>UAAGGGGGUCAGGCACUGCAUGCUCGUUCCAGCACCAUCUGGGACU<br>GGGUACAGUACCUCCAGCCCCAGGGCCCUGACCUGCGCACCUAGCUU<br>GACAUCUCACGCACCUCCCAGAGCUGGCGCCACUGAGUAAUCCGGAC<br>CUCACCACCUCUUUUCCUUUGAGCCCAAGGCAGAGCUGGAGCUGGCG<br>CCACCCAGACAGCGUCAGGUGUGGCUGGGGUAGGUUUGGAGGUCUG<br>CCAGUUACACCAAGUCCCCUCUGAGAUUCGAUCAGGGGACUGGAUA<br>GAUUCUUUCAGGUACUCAAUCAGGAAGCU
SLC12A5 UTR-psiCHECK-2-WT	UCGCUGGGGAGCAGGCGUCUCUCCUCAGUCGGCUUGUCGCCUGCUCC<br>CCGUAUCCCAUGGCUCCUCGCCAAAGACUGAAAUUGUGGAGCUGGA<br>GGGCGCCCCCUCCCCGGAGUUUCCUCCCUGGGACAAGUGAGGGAGGA<br>GGGGGCCGAUUCUGGUUUAGGGGCCGGACCCACUGAGAGGCCCCAG<br>AGCCGCCCGUGAUGUUCCUCCCCCGUCCCCAUCUGGCAGCUCCUGUC<br>UCGCCUGAGGGACCCAGCCGCCUUCUCCGUGCUCUGGGGCCGGGCCU<br>CGCUGCUUAGCAGCGGCCUCUAGCUCCGUCUCCCGGGGACCUGGGCC<br>UGAGGGAGGGCUGGAGUCGCACGCGCUUUGUCCUUAGCGCCUGUCU<br>GCUCUCCUCUAACUAGGACCCAGGGCCUUUGGCUUCCCCAGCUCAUC<br>CUUGGCCCUUCCGCUCCACCAGCCUGGUCUGAGGCGUGCUCUGUCCU<br>UAGAGAAGGCGCGGUGGCCGGGUUCCCUUCCCCUAGGGCACAUUAC<br>UAAGGGGGUCAGGCACUGCAUGCUCGUUCCAGCACCAUCUGGGACU<br>GGGUACAGUACCUCCAGCCCCAGGGCCCUGACCUGCGCACCUAGCUU<br>GACAUCUCACGCACCUCCCAGAGCUGGCGCCACUGAGUAAUCCGGAC<br>CUCACCACCUCUUUUCCUUUGAGCCCAAGGCAGAGCUGGAGCUGGCG<br>CCACCCAGACAGCGUCAGGUGUGGCUGGGGUAGGUUUGGAGGUCUG<br>CCAGUUACACCAAGUCCCCUCUGAGAUUCGAUCAGGGGACUGGAUA<br>GAUUCUUUCAGGUACUCAAUCAGGAAGCU

The labeled red nucleotides were changed into its complementary nucleotides in the mutant cohort.

Table S3. The primers of RT-PCR.

Gene	Primer（5'→3'）
TWIST1-mRNA-F	GGACAAGCTGAGCAAGATTCA
TWIST1-mRNA-R	CGGAGAAGGCGTAGCTGAG
TWIST1-3'UTR specific-F	AGCCACTGAAAGGAAAGGCAT

TWIST1-3'UTR specific-R	AGCAGGTATTTACCACCAACTTA
ZFHX4-mRNA-F	GGAGAACTGTGGGCAGAGAG
ZFHX4-mRNA-R	TCTCTGCTCCTCCTGTTC
ZFHX4-3'UTR specific-F	CAACTTCCACCACCCAGTGA
ZFHX4-3'UTR specific-R	ACCTTCTGTTACTTTGGAATGAATC
SLC12A5-mRNA-F	GCAGGAGCCATGTACATCCT
SLC12A5-mRNA-R	CCATGCAGGTGAGCACACA
SLC12A5-3'UTR specific-F	CTCTCCTCAGTCGGCTTGTC
SLC12A5-3'UTR specific-F	GGCCCTGGGTCCTAGTTAGA

Table S4. Clinicopathologic parameters and expression of TWIST1/SLC12A5/ZFHX4 in 92 of LUAD patients

Variable	Cases(n)	%
**Ages**		
＜60	51	55.4
≥60	41	44.6
**Sex**		
Male	67	72.8
Female	25	
**Degree of differation**		
Well	25	27.2
Moderate	20	21.7
Poor	47	51.1
**Tumor size**		
≤3cm	34	37.0
＞3cm	58	63.0
**Lymph node metastasis**		
No	50	54.3
Yes	42	45.6
**Pleural invasion**		
No	67	72.8
Yes	25	34.7
**TNM staging**		
I	34	37.0
II	25	26.9
III	33	35.9
**TWIST1 expression**		
High	71	77.2
Low	21	22.8
**SLC12A5 expression**		
High	61	66.3
Low	31	33.7
**ZFHX4 expression**		
High	63	68.5
Low	29	31.5

Expression of TWIST/SLC12A4/ZFHX4 was normalized to paired normal tissue. High expression was defined as tumor/non-tumor>1.

Supplementary figures

Figure S1. Expression profile of TWIST1 in LUAD cancer cell lines by RT-PCR analysis

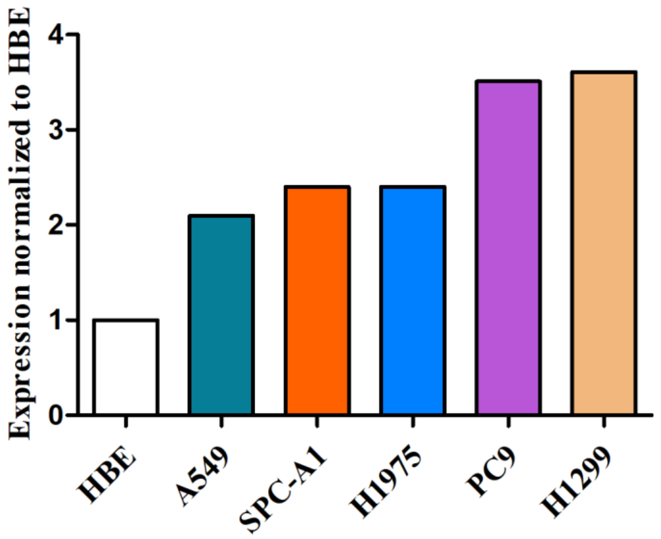

Figure S2. Overexpression of specific 3'UTR regions confirmed by q-PCR

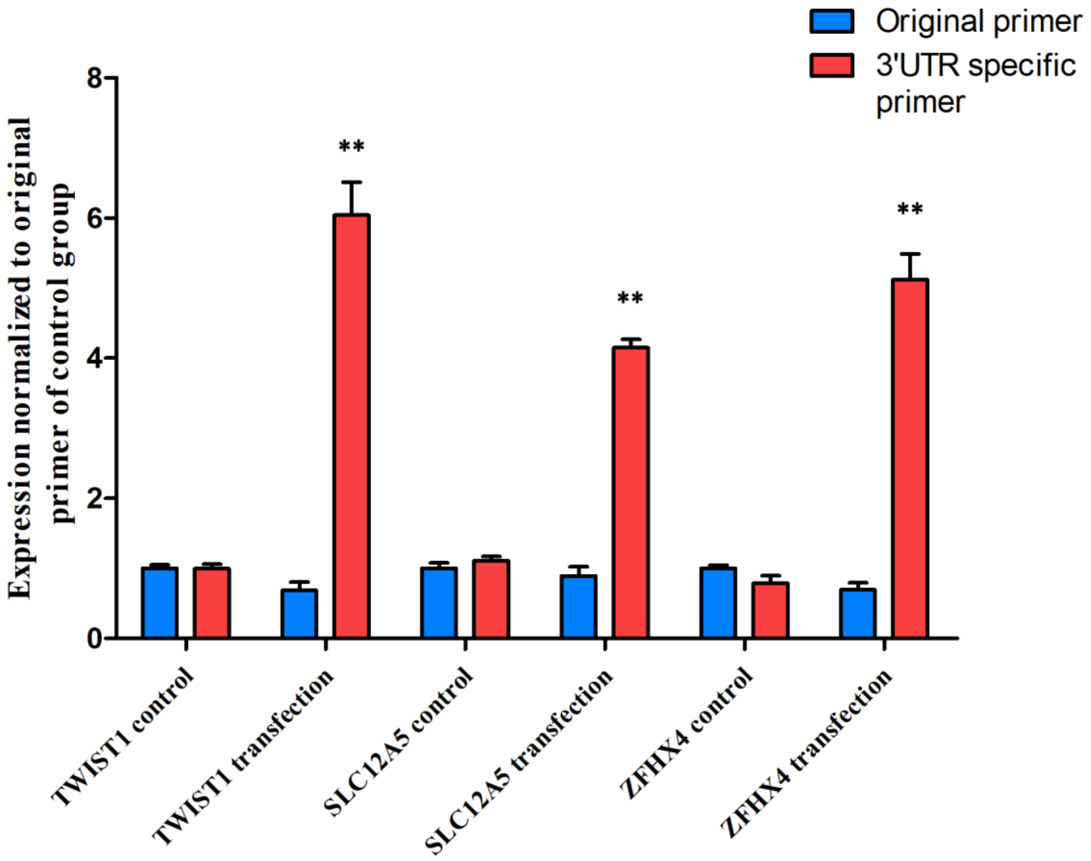

Figure S3. Biological functions of the cDNAs of TWIST1 ceRNET in A549. (A-B) Effects of TWIST1-ceRNET cDNA on proliferative capacity evaluated by 5-ethynyl-2'-deoxyuridine (EdU) proliferation assay (A) and Cell Counting Kit-8 (CCK-8) assay (B). (C) Effects of TWIST1-ceRNET cDNA on migration and invasion capacity evaluated by transwell assay and matrigel assay All data are mean ± s.d. The data statistical significances were assessed by Student's t-test compared to the NC group. *P < 0.05, **P < 0.001.

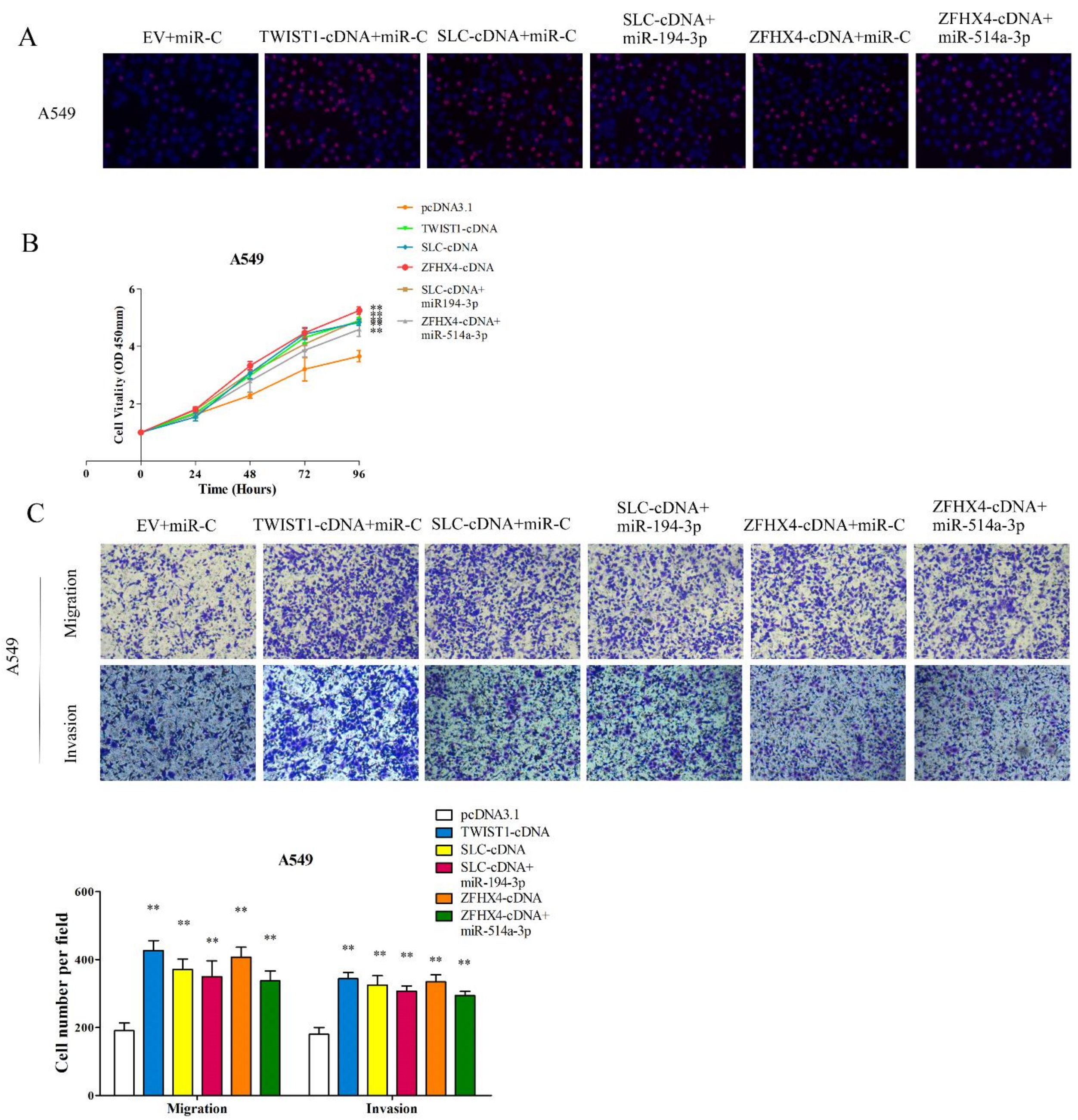

Figure S4. Q-PCR results of the RIP based on Ago2 showed that SLC12A5 and ZFHX4 can compete with the TWIST1 transcript for the binding of miRNAs in H1299 cells

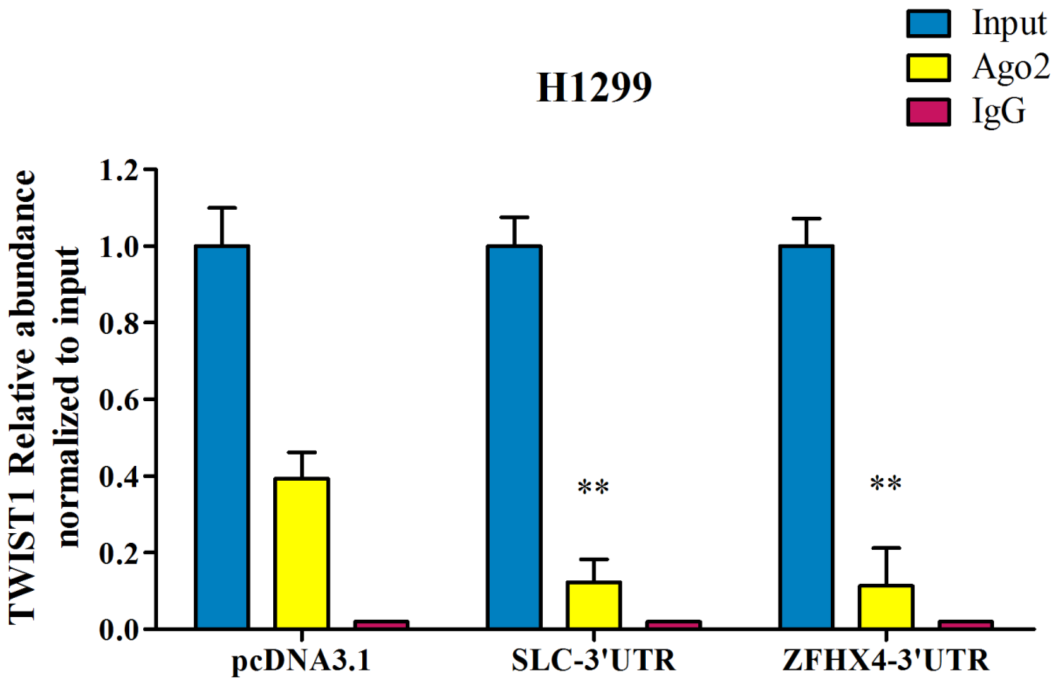

Figure S5. Q-PCR results of the RIP based on Ago2 showed that SLC12A5 and ZFHX4 can compete with the TWIST1 transcript for the binding of miRNAs in A549 cells

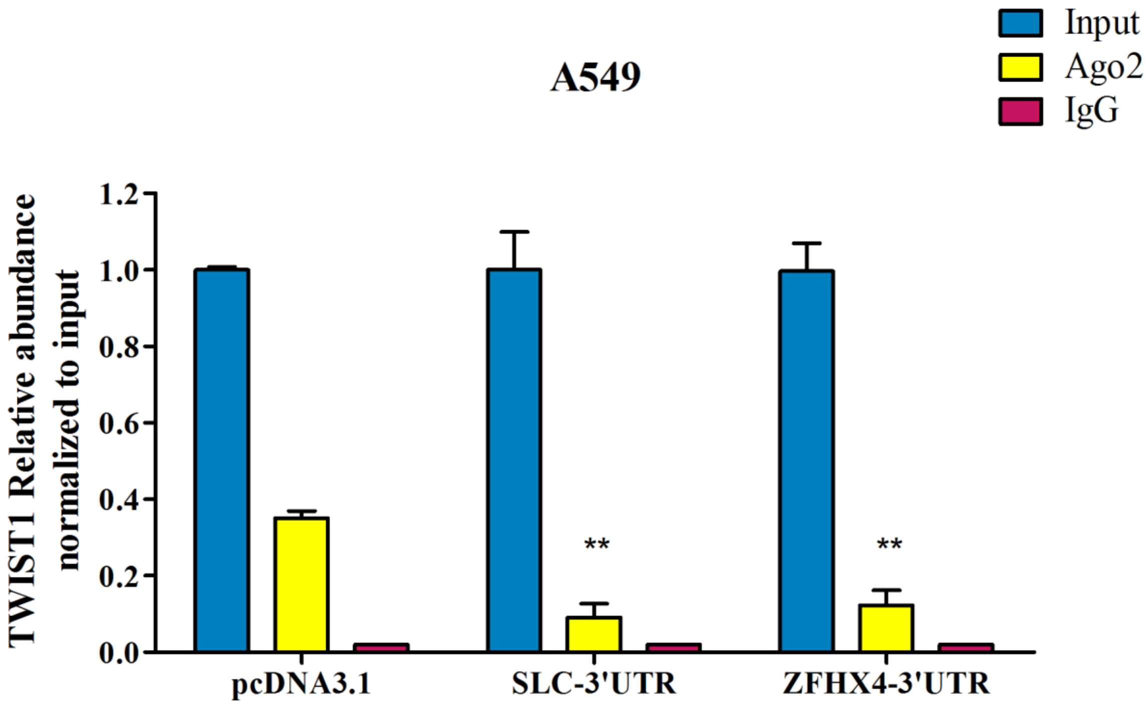

Supplement: Supplementary file 1 — supplementary materials [file 41389_2019_167_MOESM1_ESM.pdf]
